# Supplementary material for: Characterization of a novel type III CRISPR-Cas effector provides new insights into the allosteric activation and suppression of the Cas10 DNase
Source: Cell Discov. 2020 May 12;6:29. doi: 10.1038/s41421-020-0160-4 (PMC7214462; doi:10.1038/s41421-020-0160-4)
Supplement: Supplementary file 1 — Supplemental Figures and Tables [file 41421_2020_160_MOESM1_ESM.pdf]

Supplemental data to
Characterization of a novel type III CRISPR-Cas effector provides new insights into the allosteric activation and suppression of the Cas10 DNase

Jinzhong Lin[1], Mingxia Feng[1,3] Heping Zhang[4] and Qunxin She[1,2]

[1] Archaea Centre, Department of Biology, University of Copenhagen, Ole Maaløes Vej 5, DK-2200 Copenhagen N, Denmark

[2] Microbial Technology Institute and State Key Laboratory of Microbial Technology, Shandong University, 72 Binhai Road, Jimo, Qingdao, Shandong, 266237, China

[3] College of Life Science and Technology, Huazhong Agricultural University, Wuhan, China

[4] Key Laboratory of Dairy Biotechnology and Engineering, Inner Mongolia Agricultural University, Hohhot, 010018, China

Correspondence. Jinzhong Lin, email: jinzhong.lin@bio.ku.dk; Qunxin She, email: shequnxin@sdu.edu.cn

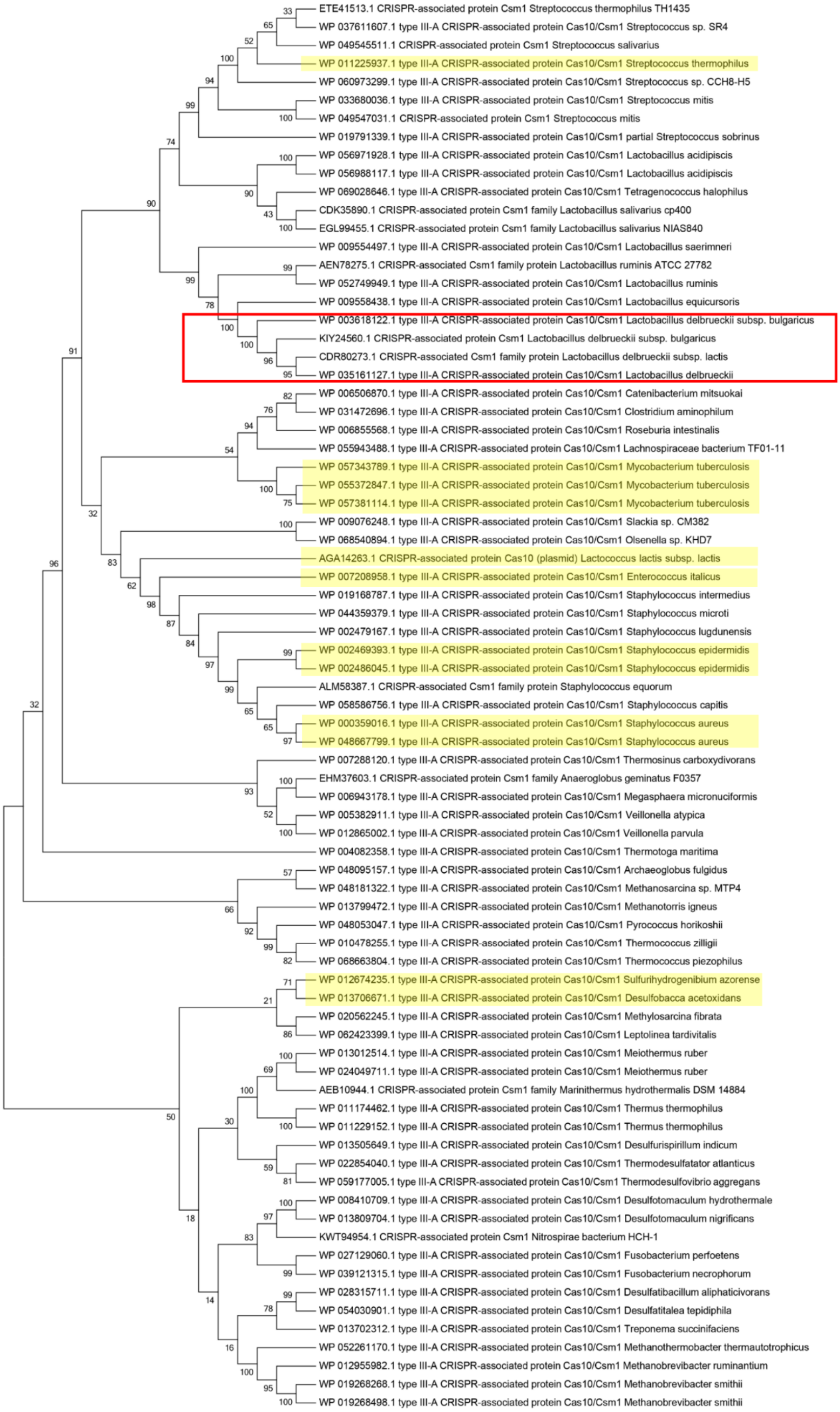

Supplementary Fig. S1 The phylogenetic tree of the type III-A CRISPR-Cas system signature protein Cas10/Csm1 using MUSCLE method in MEGA5[1]. *L. delbrueckii* subsp. was highlighted by the red line box. The

characterized type III-A system were highlighted by yellow background.

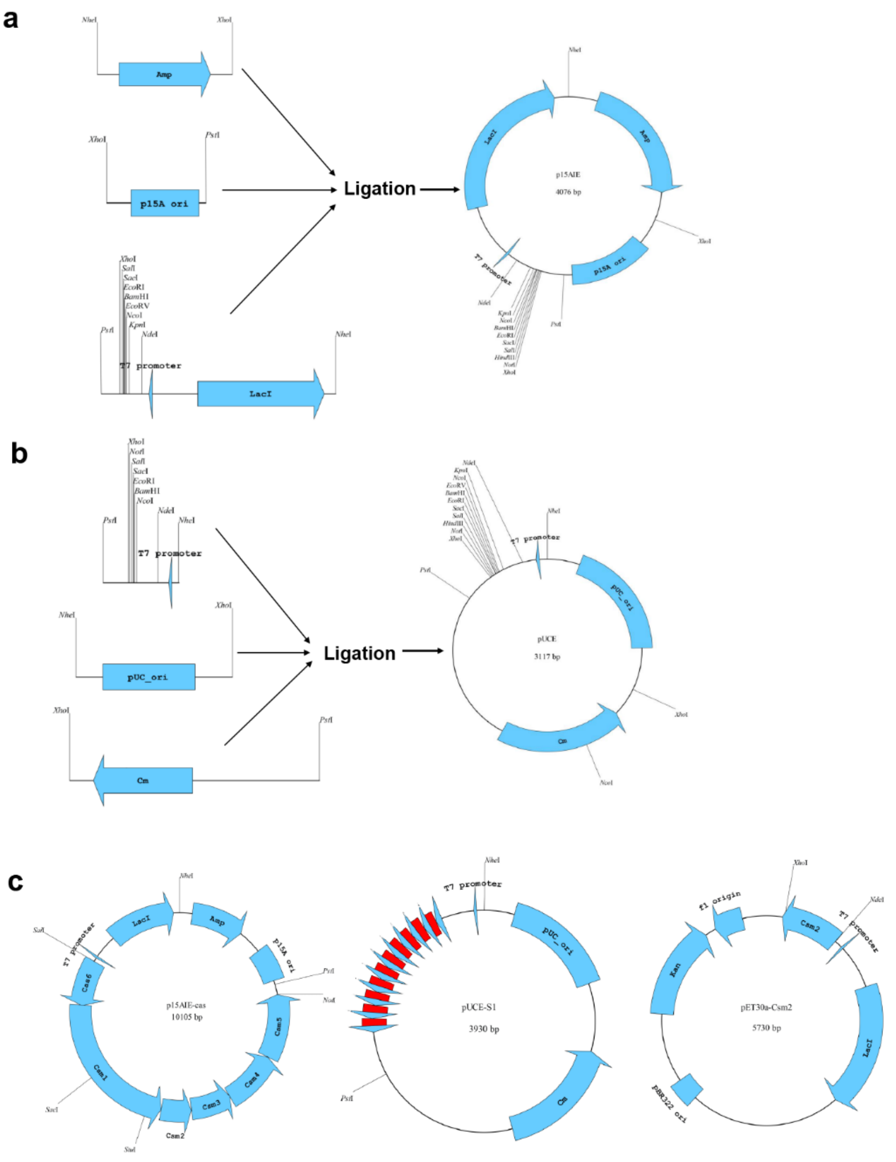

Supplementary Fig. S2 Construction of expression vectors and plasmids.

(**a**) Construction of CRISPR-Cas cassette gene expression vector p15AIE, the replicon of p15A, ampicillin resistance gene (*Amp*) and gene expression/regulation elements (including T7 promoter, multiple cloning sites, T7 transcriptional terminator and regulation gene *LacI*) were amplified from plasmids pTRKH2, pUC19 and pET30a, respectively. Then, these three PCR products were ligated together in a triple ligation, yielding the expression vector p15AIE.

(**b**) Construction of crRNA expression vector pUCE. The replicon of pUC, chloramphenicol resistance gene (Cm) and gene expression elements (including T7 promoter, multiple cloning sites, T7 transcriptional terminator) were amplified from plasmids pUC19, pCI372, and pET30a, respectively. Then, these three DNA fragments were ligated together in a triple ligation, resulting in the expression vector pUCE.

(**c**) DNA fragment covering the *cas6-cas10-csm2-csm3-csm4-csm5* gene cassette was amplified from genome of *L. delbrueckii* subsp. *bulgaricus* ND04. The PCR product was ligated to plasmid vector p15AIE, yielding p15AIE-cas. For construction of plasmid pUCE-S1, fusion PCR amplification was performed to generate the multiply 36 nt length repeats interspaced by multiply identical 40 nt spacer S1, then the PCR products of ~1 kb were recovered by agarose electrophoresis and the OMEGA gel-purification kit. The obtained DNA fragments were cloned into a blunt ligation vector pJET1.2/blunt and confirmed by DNA sequencing, then the target DNA fragment was amplified and cloned into plasmid pUCE to generate the plasmid pUCE-S1 carried ten identical tandem copies of spacer S1. Finally, *csm2* gene was amplified from strain ND04 genome, the yielding PCR products were cloned into pET30a expression vectors to generate pET30a-Csm2.

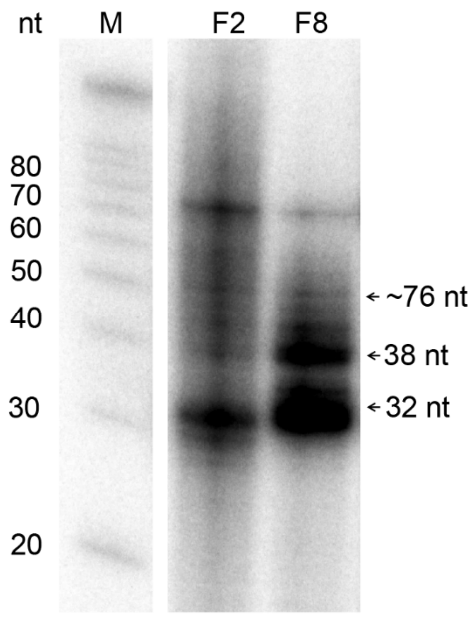

Supplementary Fig. S3 Northern blot analysis of crRNA within the LdCsm effector complexes. RNAs were extracted from the SEC-purified LdCsm samples. M: RNA size ladder.

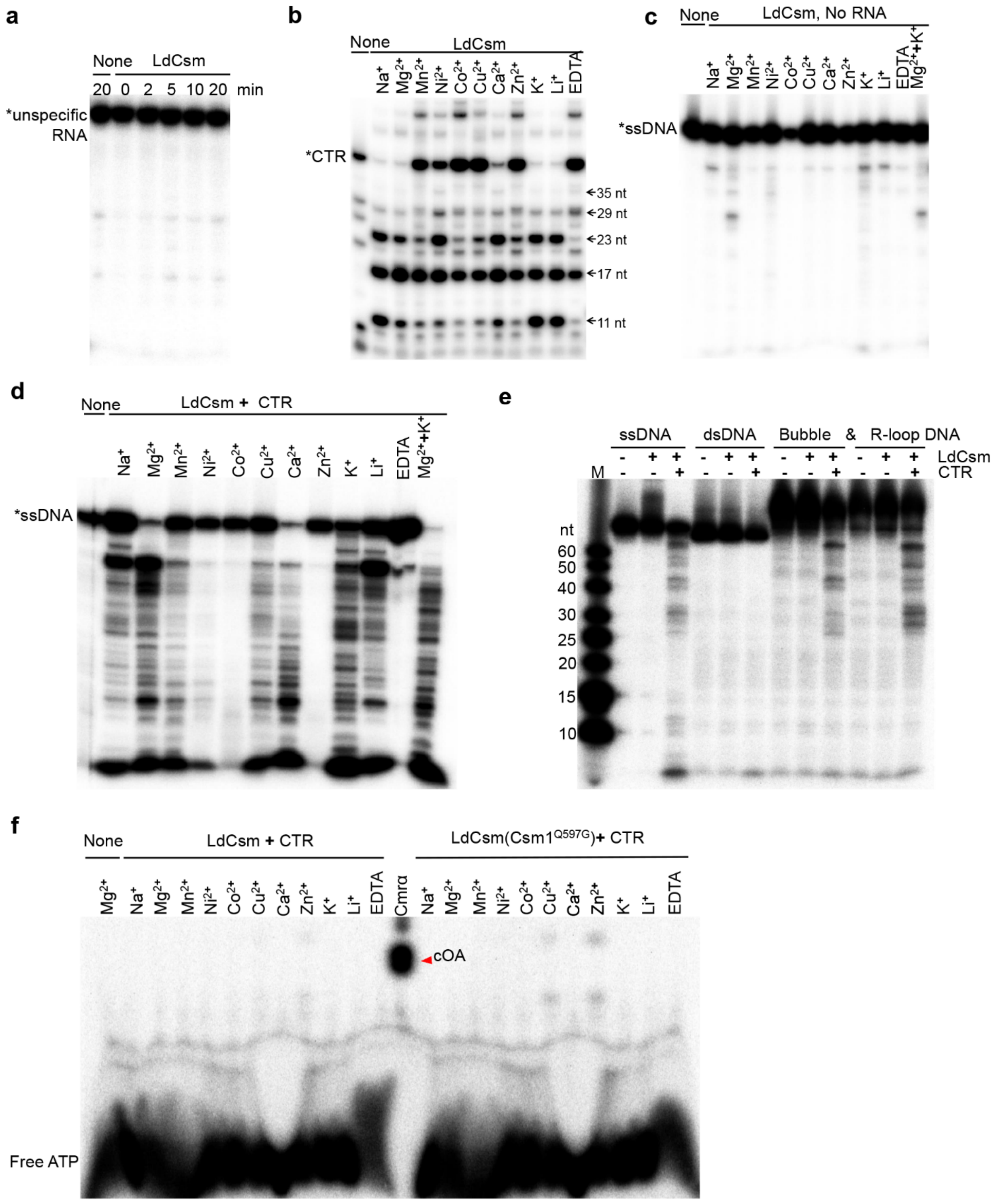

Supplementary Fig. S4 Biochemical characterization of the LdCsm effector complexes**.**

(**a**) No non-homologous RNA cleavage by LdCsm. 50 nM of S10, a non-homologous RNA, was incubated with or without 50 nM of the LdCsm for the indicated time, then, the samples were analyzed by denaturing PAGE.

(**b**) Target RNA cleavage required cations. 50 nM of S1-46 target RNA (CTR) substrates were incubated with 50 nM of LdCsm addition with different ions for 10 min, then, the samples were analyzed by denaturing PAGE. The

final concentrations of ions (Mg^{2+}, Mn^{2+}, Ni^{2+}, Cu^{2+}, Co^{2+}, Ca^{2+} and Zn^{2+}) were 10 mM, the final concentrations of Na^{+}, K^{+}, Li^{+} and EDTA were 50 mM, all the samples also contain an extra 25 mM Na+ from storage buffer for storage of LdCsm complex.

(**c**) No ssDNase activity without target RNA. 50 nM of S10-60 ssDNA substrates and 50 nM of LdCsm were incubated without any RNA in different ions for 10 min, then, the samples were analyzed by denaturing PAGE. The concentrations of ions used were identical to that used in (b).

(**d**) Target RNA-activated ssDNA cleavage required cations. 50 nM of S10-60 ssDNA substrates and 50 nM of LdCsm were incubated with 500 nM of CTR in different ions for 10 min, then, the samples were analyzed by denaturing PAGE. The concentrations of ions used were identical to that used in (b).

(**e**) LdCsm cleaved ssDNA and ssDNA region of bubble DNA and R-loop DNA. ssDNA, dsDNA, bubble DNA and R-loop DNA together with (+) or without (-) 10 nM of the LdCsm in the presence (+) or absence (-) of 500 nM of CTR were incubated for 10 min, the samples were analyzed by denaturing PAGE.

(**f**) No cOA generated by LdCsm. 2 nM α32P-ATP and 100 nM cold ATP were incubated with 50 nM LdCsm or LdCsm(Csm1^{Q597G})complex in the presence of 500 nM CTR for 120 min in different ions, then the samples were analyzed by denaturing PAGE. The concentrations of ions used were identical to that used in (b).

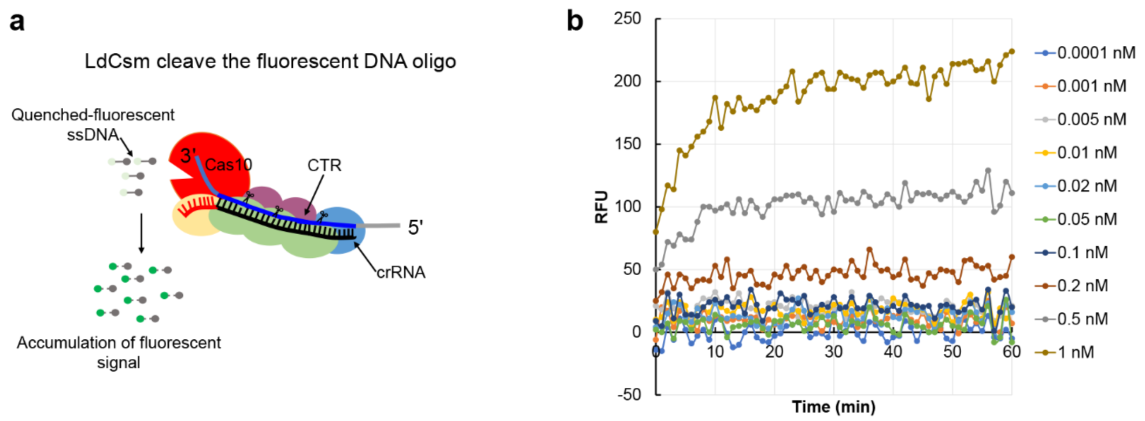

Supplementary Fig. S5 The minimal concentration of target RNA requirement for the ssDNase activity of LdCsm. (**a**) Schematic of LdCsm RNA detection approach using a quenched fluorescent ssDNA oligo. (**b**) Quantified time-course fluorescence signal generated by LdCsm with varying concentrations of S1-46 RNA (CTR). Reactions (in total 20 μl) contain 500 nM of the FAM-poly-16T-BHQ1, varying concentrations of CTR, 50 nM of Csm effector.

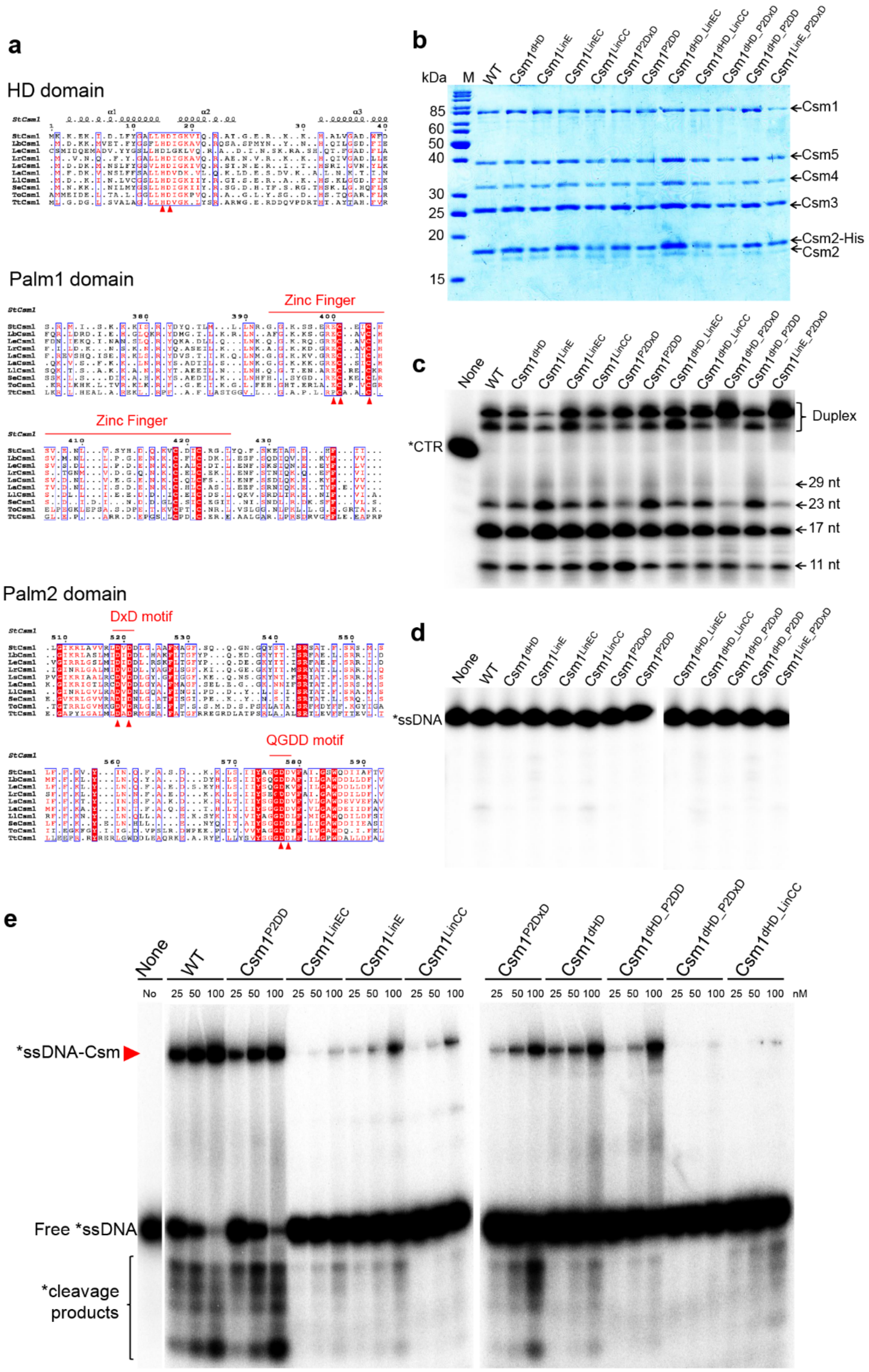

Supplementary Fig. S6 Effect of LdCsm1 mutations on the target RNA cleavage, ssDNA cleavage and binding of LdCsm.

(**a**) Conserved residues in LdCsm1. Ten Csm1 homologues: *Lactobacillus bulgaricus* (Lb), *Streptococcus*

thermophilus (St), *Lactobacillus equicursoris* (Le), *Lactobacillus ruminis* (Lr), *Lactobacillus salivarius* (Ls), *Lactobacillus acidipiscis* (La), *Lactococcus lactis* subsp. *lactis* (Ll), *Staphylococcus epidermidis* (Se), *Thermococcus onnurineus* (To) and *Thermus thermophilus* (Tt) were selected and aligned using MEGA5[1] and visualized using ESPript 3[2]. Some highly conserved residues marked with red arrows were chosen to produce the LdCsm1 variants.

(**b**) Coomassie blue-stained SDS-PAGE of protein components of the LdCsm1 mutated complexes, M: protein mass marker.

(**c**) Target RNA cleavage of LdCsm1 mutated variants. 50 nM of S1-46 target RNA (CTR) substrates were incubated with 50 nM of the indicated Csm complexes for 10 min, then, the samples were analyzed by denaturing PAGE. Duplex: Duplex of crRNA and substrate.

(**d**) No unspecific ssDNA cleavage without target RNA. 50 nM of S10-60 ssDNA substrates were incubated with 50 nM the indicated LdCsm1 mutated variants in the absence of target RNA for 10 min and the samples were analyzed by denaturing PAGE.

(**e**) Effect of LdCsm1 mutations on the ssDNA binding of LdCsm. 5 nM of S10-60 ssDNA substrates were incubated with the indicated concentration Csm in the presence of 500 nM of CTR for 3 min and then the samples were analyzed by non-denaturing PAGE, Red arrows represent the Csm-ssDNA binding complexes.

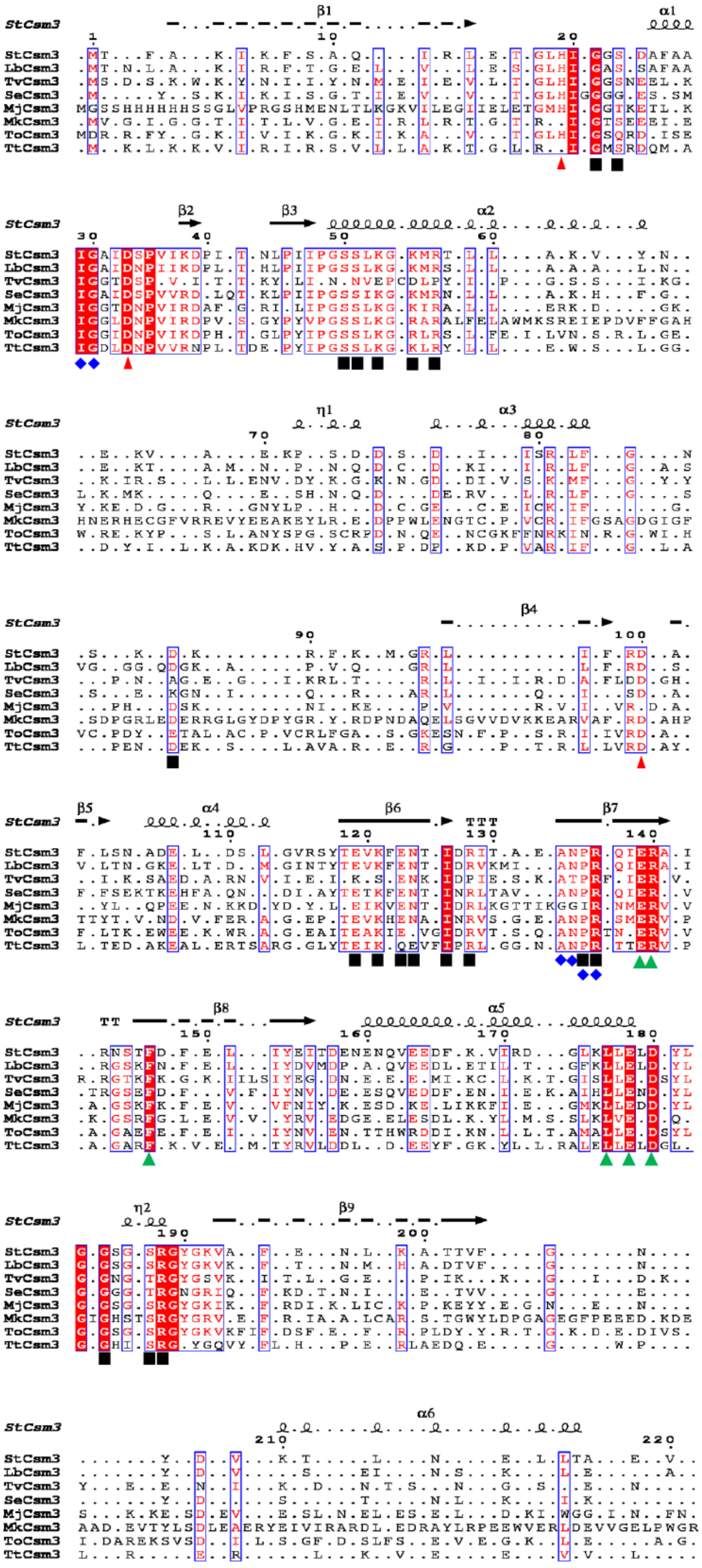

Supplementary Fig. S7 Conserved residues in LdCsm3. Eight Csm3 homologues: *Lactobacillus bulgaricus* (Lb), *Streptococcus thermophilus* (St), *Thermoplasma volcanium* (Tv), *Staphylococcus epidermidis* (Se), *Methanocaldococcus jannaschii* (Mj), *Methanopyrus kandleri* (Mk), *Thermococcus onnurineus* (To) and *Thermus thermophilus* (Tt) were selected and aligned using MEGA5[1] and visualized using ESPript 3[2]. Some highly conserved residues were chosen to produce 5 LdCsm3 variants as indicated below: H20A (Csm3^{H20A}), D34A (Csm3^{D34A}), D106A (Csm3^{D106A}), the double substitutions H20/D34A (Csm3$^{H20/D34A}$) and D34/D106A (Csm3$^{D34/D106\text{-}A}$). The amino acid residues marked with red arrows were selected to construct these variants. The amino acid residues involve crRNA binding, target RNA binding and structural function were marked with black squares, blue rhombuses and green triangles, respectively.

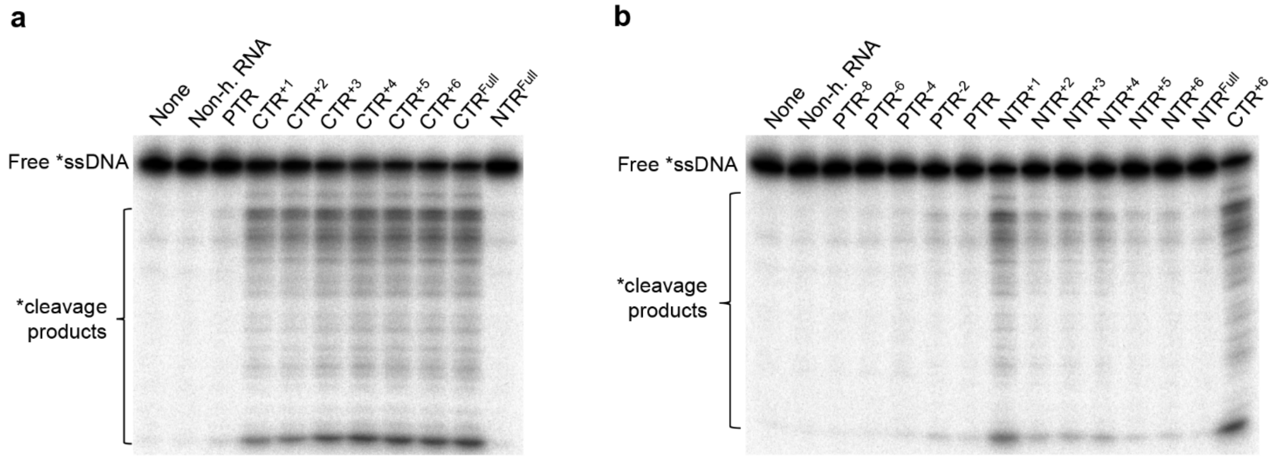

Supplementary Fig. S8 DNase activity of LdCsm by target RNA involves activation and deactivation.

(**a**) CTR activates the LdCsm DNase. (**b**) NTR mediates autoimmunity avoidance by deactivation. Reactions were set up with 5 nM S10-60 ssDNA, 100 nM of LdCsm and 500 nM target RNA. After addition of one of target RNAs, the mixture was incubated at 37 °C for 3 min. Samples were then analyzed by denaturing PAGE. The percentage ssDNA cleaved of LdCsm facilitated by each of these target RNAs were estimated by image quantification of bands and showed in Fig. 5, using the accessory analysis tool equipped with a Typhoon FLA 7000. Results of average of three independent assays are shown with bars representing the mean standard deviation (± SD).

Supplementary Table S1 Nucleic acid oligos and substrates used in this study.

ssDNA and RNA	Sequence (5'-3')	Description
**oligo**		
p15A-F	AGCCTGCAGGCGGTTTGCGTATTGGCTAG	For plasmid p15AIE construction
p15A-R	GGCCCTCGAGAAAAAAGGCTGCACCGG	For plasmid p15AIE construction
AMP-F	CTAGGCTAGCGACGAAAGGGCCTCGTGATAC	For plasmid p15AIE construction
Amp-R	GGCCCTCGAGCAGCAGATTACGCGCAG	For plasmid p15AIE construction
LacI-F	CTAGGCTAGCCACCGGAAGGAGCTGACTG	For plasmid p15AIE construction
T7-F	CTAGGCTAGCTCCGGCGTAGAGGATCGAG	For plasmid pUCE construction
T7-R	AGCCTGCAGATCCGGATATAGTTCCTCC	For plasmids p15AIE and pUCE construction
Cm-F	AGCCTGCAGCTTAAGGAACGTACAGACGG	For plasmid pUCE construction
Cm-R	CGCCTCGAGCATGTGAGGCATTTTCGCTC	For plasmid pUCE construction
pUC_ori-F	CTAGGCTAGCCTGTCAGACCAAGTTTACTC	For plasmid pUCE construction
pUC_ori-R	GGCCCTCGAGCATTAATGAATCGGCCAACGC	For plasmid pUCE construction
SalI-Cas6-F	AGGGTCGACGTGCAGAAGATAAGTTTAGTTTG	For plasmid p15AIE-Cas construction
NotI-Csm5-R	ATGGGCGGCCGCTCATTTCTTTTCTTTGATGGT G	For plasmid p15AIE-Cas construction
Csm2-F	GAATTCCATATGTACGGAAGTCCACGTCC	For plasmid pET30a-Csm2 construction
Csm2-R	GGCCCTCGAGATCCTTTCCGCCGTAATACT	For plasmid pET30a-Csm2 construction
Re-S1-F	GCTAACAACTTATCTCCGCTAGAAGGAGACGAG AACTTCAAAGCTTA	For plasmid pUCE-S1 construction
Re-S1-R	GTTCTCGTCTCCTTCTAGCGGAGATAAGTTGTTA GCTGTTAAGTCTGGT	For plasmid pUCE-S1 construction
S1-R1	AGCTGTTAAGTCTGGTTTCCCTCCAGGGTATCT AAGCTTTGAAGTT	For plasmid pUCE-S1 construction
pBR322&Kan-F	CCGGAATTCGTTTACAATTTCAGGTGGC	For plasmid pBad construction
pBR322&Kan-R	CCGGGAGCTCTGAGAGCCTTCAACCCAGTC	For plasmid pBad construction
PBad-F	CCGGGAGCTCAGAAACCAATTGTCCATATTG	For plasmid pBad construction
PBad-R	CGTTTCACTCCATCCAAAAAAACG	For plasmid pBad construction
MCS-T-F	TTTTGGATGGAGTGAAACGATGCACCATCATCAT CATCAT	For plasmid pBad construction
MCS-T-R	CCGGAATTCCAACAGAACCGTTTCTACTC	For plasmid pBad construction
eGFP-F	CCGCCCATGGCAAGGGCGAGGAGCTG	For plasmid pBad-G construction
eGFP-R	CGGGGTACCAATCTCGATGTTGTGGCGGATC	For plasmids pBad-G and pBad-CTR or pBad-NTR construction
S1-F	CCGCCCATGGAAGTCTGGTTTCCCTCCAGGGTA	For plasmids pBad-CTR and

	TCTAAGCTTTG	pBad-NTR construction
CTR-GFP-F	CCCTCCAGGGTATCTAAGCTTTGAAAAAAAAAG CAAGGGCGAGGAGCTG	For plasmid pBad-CTR construction
NTR-GFP-F	CTCCAGGGTATCTAAGCTTTGAAGTTCTCGTCA GCAAGGGCGAGGAGCTG	For plasmid pBad-NTR construction
Csm1-H15D16A-F	GTTTCTGGCTGCTATTGGTAAAGCCGTCCAAAG	For LdCsm1 H15D16-A mutation
Csm1-H15D16A-R	TACCAATAGCAGCCAGAAACGATCCGTAGAAGG	For LdCsm1 H15D16-A mutation
Csm1-D599D600A-F	CACAAGGTGCTGCTGCCTTTATTTTAGGTGCCT G	For LdCsm1 D599D600-A mutation
Csm1-D599D600A-R	TAAAGGCAGCAGCACCTTGTGAATAGATAATAC	For LdCsm1 D599D600-A mutation
Csm3-H20A-F	GCGGCCTGGCTATTGGCGCAAGTTCAGCTTTTG	For LdCsm3 H20A and H20/D34-A mutations
Csm3-H20A-R	GCGCCAATAGCCAGGCCGCTTTCCAGTACC	For LdCsm3 H20A and H20/D34-A mutations
Csm3-D34A-F	GGTGCGATCGCCAACCCGATAATTAAGGATCC	For LdCsm3 D34A mutation
Csm3-D34A-R	ATCGGGTTGGCGATCGCACCAATAGCCGCA	For LdCsm3 D34A mutation
Csm3-D106A-F	TATTTAGAGCCAGTGTTCTGACTAATGGCA	For LdCsm3 D106A and D34/D106-A mutations
Csm3-D106A-R	AGAACACTGGCTCTAAATAAAAGCCGCCCT	For LdCsm3 D106A and D34/D106-A mutations
SacI-Csm1-F	CTATGCTGAGCTCTTCCAGAG	For LdCsm1 Palm1 and Palm 2 mutations
SacI-Csm1-R	CTCTGGAAGAGCTCAGCATAG	For LdCsm1 H15D16-A mutation
StuI-Csm1-F	CGAGGTAAGGCCTTCATTTAC	For LdCsm3 D34A mutation
StuI-Csm1-R	GTAAATGAAGGCCTTACCTCG	For LdCsm1 Palm1 and Palm 2 mutations
KpnI-Csm4-R	GACAGTGGTACCGCGTATAAGC	For LdCsm3 D34A mutation
Csm1N-E415C416A-F	AAAAGAGTGGCCGGGCGGCTGCAGTTTGTCAT AGCGTTATG	For LdCsm1 E415C416-A mutation
Csm1N-E415C416A-R	TATGACAAACTGCAGCCGCCCGGCCACTCTTTT TGCCAAAG	For LdCsm1 E415C416-A mutation
Csm1N-Q597G-F	GAGTATTATCTATTCAGGAGGTGATGATGCCTTT ATTTTAGG	For LdCsm1 Q597G mutation
Csm1N-Q597G-R	ATAAAGGCATCATCACCTCCTGAATAGATAATACT CAGATG	For LdCsm1 Q597G mutation
Csm1N-E415A-F	AAAAGAGTGGCCGGGCGTGTGCAGTTTGTCATA GCGTTATG	For LdCsm1 E415A mutation
Csm1N-E415A-R	TATGACAAACTGCACACGCCCGGCCACTCTTTT	For LdCsm1 E415A mutation

	TGCCAAAG	
Csm1N-C416/419A-F	GGAGGCTGCAGTTGCTCATAGCGTTATGAATCTCC	For LdCsm1 C416/419-A mutation
Csm1N-C416/419A-R	TATGAGCAACTGCAGCCTCCCGGCCACTCTTTTTGC	For LdCsm1 C416/419-A mutation
Csm1N-D541/543A-F	ATGATCGCTATTGCTGACCTCCATGCCAAATTCT	For LdCsm1 D541/543-A mutation
Csm1N-D541/543A-R	GAGGTCAGCAATAGCGATCATCAAAGAAGCAAG	For LdCsm1 D541/543-A mutation
**Nucleic acid substrates**		
S10 RNA	AUAGAAUGCCCCCAUUAUACAAUAUCUACGUUUUAGAUGAAAAAAA	Non-homologous RNA
S1-40 (PTR)	UGUUAAGUCUGGUUUCCCUCCAGGGUAUCUAAGCUUUGAA	Target RNA Lacking any 3' anti-tag
S1-46 (CTR)	UGUUAAGUCUGGUUUCCCUCCAGGGUAUCUAAGCUUUGAAAAAAAA	Target RNA with noncomplementary 3' anti-tag with crRNA
S1-48 (NTR)	UGUUAAGUCUGGUUUCCCUCCAGGGUAUCUAAGCUUUGAAGUUCUCGU	Target RNA with complementary 3' anti-tag with crRNA
S10-60 ssDNA	ACTATAGGGAGAATAGAATGCCCCCATTATACAATATCTACGTTTTAGATGACCCCCCCC	Non-homologous ssDNA substrate
PTR^{-2}	GGUGUUAAGUCUGGUUUCCCUCCAGGGUAUCUAAGCUUUG	A PTR Lacking double nucleotides in 3'-end
PTR^{-4}	GGUGUUAAGUCUGGUUUCCCUCCAGGGUAUCUAAGCUU	A PTR Lacking four nucleotides in 3'-end
PTR^{-6}	GGUGUUAAGUCUGGUUUCCCUCCAGGGUAUCUAAGC	A PTR Lacking six nucleotides in 3'-end
PTR^{-8}	GGUGUUAAGUCUGGUUUCCCUCCAGGGUAUCUAA	A PTR Lacking eight nucleotides in 3'-end
CTR^{+1}	GGUGUUAAGUCUGGUUUCCCUCCAGGGUAUCUAAGCUUUGAAA	Cognate target RNA with single A in 3' anti-tag
CTR^{+2}	GGUGUUAAGUCUGGUUUCCCUCCAGGGUAUCUAAGCUUUGAAAA	Cognate target RNA with double A in 3' anti-tag
CTR^{+3}	GGUGUUAAGUCUGGUUUCCCUCCAGGGUAUCUAAGCUUUGAAAAA	Cognate target RNA with triple A in 3' anti-tag
CTR^{+4}	GGUGUUAAGUCUGGUUUCCCUCCAGGGUAUCUAAGCUUUGAAAAAA	Cognate target RNA with four A in 3' anti-tag
CTR^{+5}	GGUGUUAAGUCUGGUUUCCCUCCAGGGUAUCUAAGCUUUGAAAAAAA	Cognate target RNA with five A in 3' anti-tag
CTR^{+6}	GGUGUUAAGUCUGGUUUCCCUCCAGGGUAUCUAAGCUUUGAAAAAAAA	Cognate target RNA with six A in 3' anti-tag

CTR^{Full}	GGUGUUAAGUCUGGUUUCCCUCCAGGGUAUC UAAGCUUUGAAAAAAAAA	Cognate target RNA with eight A in 3' anti-tag
NTR^{+1}	GGUGUUAAGUCUGGUUUCCCUCCAGGGUAUC UAAGCUUUGAAG	Non-Cognate target RNA with single nucleotide in 3' anti-tag
NTR^{+2}	GGUGUUAAGUCUGGUUUCCCUCCAGGGUAUC UAAGCUUUGAAGU	Non-Cognate target RNA with double nucleotides in 3' anti-tag region
NTR^{+3}	GGUGUUAAGUCUGGUUUCCCUCCAGGGUAUC UAAGCUUUGAAGUU	Non-Cognate target RNA with triple nucleotides in 3' anti-tag
NTR^{+4}	GGUGUUAAGUCUGGUUUCCCUCCAGGGUAUC UAAGCUUUGAAGUUC	Non-Cognate target RNA with four nucleotides in 3' anti-tag
NTR^{+5}	GGUGUUAAGUCUGGUUUCCCUCCAGGGUAUC UAAGCUUUGAAGUUCU	Non-Cognate target RNA with five nucleotides in 3' anti-tag
NTR^{+6}	GGUGUUAAGUCUGGUUUCCCUCCAGGGUAUC UAAGCUUUGAAGUUCUC	Non-Cognate target RNA with six nucleotides in 3' anti-tag
NTR^{Full}	GGUGUUAAGUCUGGUUUCCCUCCAGGGUAUC UAAGCUUUGAAGUUCUCGU	Non-Cognate target RNA with eight nucleotides in 3' anti-tag

Supplementary Table S2 LdCsm1 and LdCsm3 mutations utilized in this study.

RNP Name	Description
Csm1^{dHD}	A HD domain mutant carrying H15A and D16A substitutions
Csm1^{LinE}	A Linker domain mutant carrying E415A substitution
Csm1^{LinEC}	A Linker domain mutant carrying E415A and C416A substitution
Csm1^{LinCC}	A Linker domain mutant carrying C416A and C419A substitutions
Csm1^{P2DxD}	A Palm2 domain mutant carrying D541A and D543A substitutions
Csm1^{P2DD}	A Palm2 domain mutant carrying D599A and D600A substitutions
Csm1^{dHD_LinEC}	A Csm1 mutant carrying H15A, D16A, E415A and C416A substitutions
Csm1^{dHD_LinCC}	A Csm1 mutant carrying H15A, D16A, C416A and C419A substitutions
Csm1^{dHD_P2DxD}	A Csm1 mutant carrying H15A, D16A, D541A and D543A substitutions
Csm1^{dHD_P2DD}	A Csm1 mutant carrying H15A, D16A, D599A and D600A substitutions
Csm1^{LinE_P2DxD}	A Csm1 mutant carrying E415A, D541A and D543A substitutions
Csm1^{Q597G}	A Csm1 mutant carrying Q597G substitution
Csm3^{H20A}	A Csm3 mutant carrying H20A substitution

Csm3^{D34A}	A Csm3 mutant carrying D34A substitution
Csm3^{D106A}	A Csm3 mutant carrying D106A substitution
Csm3$^{H20/D34-A}$	A Csm3 mutant carrying H20A and D34A substitutions
Csm3$^{D34/106-A}$	A Csm3 mutant carrying D34A and D106A substitutions

References

1 Tamura, K. et al. MEGA5: molecular evolutionary genetics analysis using maximum likelihood, evolutionary distance, and maximum parsimony methods. *Mol. Biol. Evol.* 28, 2731-2739 (2011).

2 Robert, X. & Gouet, P. Deciphering key features in protein structures with the new ENDscript server. *Nucleic Acids Res.* 42, W320-324 (2014).
